# Improved outcomes over time and higher mortality in CMV seropositive allogeneic stem cell transplantation patients with COVID-19; An infectious disease working party study from the European Society for Blood and Marrow Transplantation registry

**DOI:** 10.3389/fimmu.2023.1125824

**Published:** 2023-03-07

**Authors:** Per Ljungman, Gloria Tridello, Jose Luis Piñana, Fabio Ciceri, Henrik Sengeloev, Alexander Kulagin, Stephan Mielke, Zeynep Arzu Yegin, Matthew Collin, Sigrun Einardottir, Sophie Ducastelle Lepretre, Johan Maertens, Antonio Campos, Elisabetta Metafuni, Herbert Pichler, Frantisek Folber, Carlos Solano, Emma Nicholson, Meltem Kurt Yüksel, Kristina Carlson, Beatriz Aguado, Caroline Besley, Jenny Byrne, Immaculada Heras, Fiona Dignan, Nicolaus Kröger, Christine Robin, Anjum Khan, Stig Lenhoff, Anna Grassi, Veronika Dobsinska, Nuno Miranda, Maria-Jose Jimenez, Ipek Yonal-Hindilerden, Keith Wilson, Dina Averbuch, Simone Cesaro, Alienor Xhaard, Nina Knelange, Jan Styczynski, Malgorzata Mikulska, Rafael de la Camara

**Affiliations:** ^1^ Department of Cellular Therapy and Allogeneic Stem Cell Transplantation, Karolinska Comprehensive Cancer Center, Karolinska University Hospital Huddinge, Stockholm, Sweden; ^2^ Division of Hematology, Department of Medicine Huddinge, Karolinska Institutet, Stockholm, Sweden; ^3^ European Society for Blood and Marrow Transplantation (EBMT) Data Office, Department of Medical Statistics & Bioinformatics, Leiden, Netherlands; ^4^ Hematology Department, Hospital Clínico Universitario de Valencia, Valencia, Spain; ^5^ Fundación Investigación del Hospital Clínico de la Comunidad Valenciana (INCLIVA), Instituto de Investigación Sanitaria Hospital Clínico Universitario de Valencia, Valencia, Spain; ^6^ Istituto di Ricovero e Cura a Carattere Scientifico (IRCCS) Ospedale San Raffaele, University Vita-Salute San Raffaele, Milan, Italy; ^7^ Department of Hematology, Copenhagen University Hospital, Copenhagen, Denmark; ^8^ Raisa Memorial (RM) Gorbacheva Research Institute, Pavlov University, St. Petersburg, Russia; ^9^ Department of Laboratory Medicine, Karolinska Institutet, Stockholm, Sweden; ^10^ Department of Hematology, Gazi University Faculty of Medicine, Ankara, Türkiye; ^11^ Translational and Clinical Research Institute and The National Institute for Health and Care Research (NIHR) Newcastle Biomedical Research Centre, Newcastle, United Kingdom; ^12^ Department of Hematology, Sahlgrenska University Hospital, Gothenburg, Sweden; ^13^ Department of Internal Medicine, Institute of Medicine, Sahlgrenska Academy, University of Gothenburg, Gothenburg, Sweden; ^14^ Service d’Hématologie. Hôpital Lyon Sud, Lyon, France; ^15^ Department of Hematology University Hospital Gasthuisberg, Leuven, Belgium; ^16^ Marrow Transplant Department Inst. Português de Oncologia do Porto, Porto, Portugal; ^17^ Dipartimento di Diagnostica per Immagini, Radioterapia Oncologica e Ematologia, Fondazione Policlinico Universitario Agostino Gemelli Istituto di Ricovero e Cura a Carattere Scientifico (IRCCS), Rome, Italy; ^18^ St. Anna Children’s Hospital, Department of Pediatrics and Adolescent Medicine, Medical University of Vienna, Vienna, Austria; ^19^ Department of Internal Medicine, Hematology and Oncology University Hospital Brno, Brno, Czechia; ^20^ Department of Internal Medicine, Hematology and Oncology Masaryk University, Brno, Czechia; ^21^ Haematology-oncology Unit Royal Marsden Hospital, London, United Kingdom; ^22^ Department of Hematology, Ankara University Faculty of Medicine, Ankara, Türkiye; ^23^ Department of Hematology, Uppsala University Hospital, Uppsala, Sweden; ^24^ Department of Hematology, Hospital Universitario de la Princesa, Madrid, Spain; ^25^ University Hospitals Bristol and Weston National Health Service (NHS) Foundation Trust, Bristol, United Kingdom; ^26^ Department of Haematology Nottingham University Hospital, Nottingham, United Kingdom; ^27^ Department of Hematology, Hospital Universitario Morales Meseguer, Instituto Murciano de Investigación Biosanitaria (IMIB)-Arrixaca, Universidad de Murcia, Murcia, Spain; ^28^ Clinical Haematology Department Manchester Royal Infirmary, Manchester, United Kingdom; ^29^ Department of Stem cell Transplantation, University Hospital Eppendorf, Hamburg, Germany; ^30^ Assistance Publique-Hôpitaux de Paris (APHP), Henri Mondor Hospital, Department of Hematology, Créteil, France; ^31^ Department of Haematology Leeds Teaching Hospitals NHS Trust, Leeds, United Kingdom; ^32^ Department of Hematology Skåne’s University Hospital, Lund, Sweden; ^33^ Bone Marrow Transplantation Unit, Azienda Sociosanitaria Territoriale (ASST) Papa Giovanni XXIII, Bergamo, Italy; ^34^ Department of Pediatric Hematology and Oncology, National Institute of Children’s Diseases, Comenius University, Bratislava, Slovakia; ^35^ Department of Haematology Inst. Portugues Oncologia, Lisbon, Portugal; ^36^ Department of Clinical Haematology Institut Catala d'Oncologia (ICO)-Hospital Universitari Germans Trias i Pujol, Badalona, Spain; ^37^ Istanbul Medical Faculty, Adult Hematopoietic Stem Cell Transplant Center, Istanbul University, Istanbul, Türkiye; ^38^ Blood and Bone Marrow Transplantation Department University of Cardiff, Cardiff, United Kingdom; ^39^ Pediatric Infectious Diseases Unit, Hadassah Medical Center and Faculty of Medicine, Hebrew University of Jerusalem, Jerusalem, Israel; ^40^ Pediatric Hematology Oncology, Azienda Ospedaliera Universitaria Integrata, Verona, Italy; ^41^ Hematology-Transplantation Unit, Department of Hematology: Hôpital St. Louis, Paris, France; ^42^ European Society for Blood and Marrow Transplantation (EBMT) Leiden Study Unit, European Society for Blood and Marrow Transplantation (EBMT) Data Office, Leiden, Netherlands; ^43^ Department of Pediatric Hematology and Oncology, Collegium Medicum, Nicolaus Copernicus University Torun, Bydgoszcz, Poland; ^44^ Division of Infectious Diseases, University of Genoa and Ospedale Policlinico San Martino, Genova, Italy

**Keywords:** COVID-19, allogeneic, stem cell transplantation, CMV, risk factors

## Abstract

**Introduction:**

COVID-19 has been associated with high morbidity and mortality in allogeneic hematopoietic stem cell transplant (allo-HCT) recipients.

**Methods:**

This study reports on 986 patients reported to the EBMT registry during the first 29 months of the pandemic.

**Results:**

The median age was 50.3 years (min – max; 1.0 – 80.7). The median time from most recent HCT to diagnosis of COVID-19 was 20 months (min – max; 0.0 – 383.9). The median time was 19.3 (0.0 - 287.6) months during 2020, 21.2 (0.1 - 324.5) months during 2021, and 19.7 (0.1 – 383.9) months during 2022 (p = NS). 145/986 (14.7%) patients died; 124 (12.6%) due to COVID-19 and 21 of other causes. Only 2/204 (1%) fully vaccinated patients died from COVID-19. There was a successive improvement in overall survival over time. In multivariate analysis, increasing age (p<.0001), worse performance status (p<.0001), contracting COVID-19 within the first 30 days (p<.0001) or 30 – 100 days after HCT (p=.003), ongoing immunosuppression (p=.004), pre-existing lung disease (p=.003), and recipient CMV seropositivity (p=.004) had negative impact on overall survival while patients contracting COVID-19 in 2020 (p<.0001) or 2021 (p=.027) had worse overall survival than patients with COVID-19 diagnosed in 2022.

**Discussion:**

Although the outcome of COVID-19 has improved, patients having risk factors were still at risk for severe COVID-19 including death.

## Introduction

The SARS-CoV-2 emerged at the end of 2019, and Coronavirus Disease 2019 (COVID-19) started spreading worldwide. The WHO classified COVID-19 as a pandemic on March 11, 2020. During the two years of the pandemic, several variants have emerged including Omicron variants, which started to spread in the end of 2021, and which have been the dominating variants during 2022. It was early recognized that immunocompromised patients were at a high risk for severe COVID-19 with ensuing high mortality. Allogeneic hematopoietic cell transplant recipients (allo-HCT) are prone to develop severe infections with many viruses including community-acquired respiratory viruses. This has been shown to be the case also with SARS-CoV-2. The European Society for Blood and Marrow Transplantation (EBMT) initiated a prospective case collection in February 2020 and two reports have been published about the outcome in autologous and allogeneic HCT recipients and patients having undergone CAR T cell treatment, respectively (1, 2). Both analyses showed a high mortality reaching almost 30% in HCT recipients during the first period of the pandemic. Since then, variants with higher transmissibility but lower lethality have been emerging, and many improvements in management have been implemented including easy access to diagnostic tests, antiviral drugs (remdesivir, nirmatrelvir/ritonavir, molnupiravir), and monoclonal antibodies for treatment, vaccination, and for symptomatic patients improved triage algorithms and supportive care.

This paper aimed to analyze the outcome of COVID-19 in allo-HCT patients from February 2020 to July 2022 with the aim to see if the outcome has improved and if the risk factors are the same for severe and fatal COVID-19 also during the recent Omicron period. We also added CMV serology to the risk factors with the hypothesis that CMV seropositive patients could have worse outcome compared to CMV seronegative.

## Methods

This prospective survey has merged newly collected data with previous data existing in the EBMT registry. All patients gave informed consent for their data to be included in the registry. The case record forms have changed during the pandemic as knowledge have been gained but questions included the symptoms, potential risk factors for development of lower respiratory tract disease requiring ventilatory support, treatments, the need for hospitalization, intensive care, and outcome. In addition to the COVID-19 specific forms, the EBMT registry’s so called Minimal Essential Data A (MED-A) was used to extract previously submitted data regarding baseline patient information, data regarding the underlying diagnosis, and the transplant procedure, which were used in the analysis.

Criteria for inclusion in the study were that the patient should be PCR positive for SARS-CoV-2 regardless of symptoms and have undergone an allo-HCT at any time before the diagnosis of COVID-19. From the beginning of 2021, a positive SARS-CoV-2 antigen test was also accepted for study inclusion. The Swedish central Ethical Board (EPM 2020-01731) approved the study and other approvals, if required, were obtained according to national regulations. For this analysis, patients diagnosed with SARS-CoV-2 infection before July 15, 2022 were included and patients needed to have at least six weeks of follow-up.

The analysis was split in patients diagnosed with COVID-19 during the three calendar years (2020, 2021, and 2022). The first period includes the cohort already reported (2). We also did another analysis with the aim to mimic the important phases of the pandemic: February – July 2020 representing the initial phase. Most patients in this cohort were included in our previous publication (2). August 2020 – January 2021, February 2021 – November 2021 representing the early vaccination phase during which the alfa and delta variants were dominating, and finally December 2021 – July 2022 representing the Omicron phase.

### Statistics

The main characteristics of patients were reported by descriptive statistics. Median, minimum and maximum values were used for continuous variables, while absolute and percentage frequencies were used for categorical variables. Comparisons between categorical variables were performed by the Chi square or Fisher exact test, as appropriate, while continuous variables were compared by t-test or Kruskal-Wallis test. The overall survival was estimated by using the Kaplan Meier methods, considering the death due to any cause as an event and the time from COVID-19 infection to the latest follow-up as survival time; the difference between groups was tested by the log-rank test. Univariate and multivariate risk factor analysis for overall survival were performed with the Cox regression model. Variables with a p-value < 0.2 at univariate analysis were entered into the multivariate models and selected according to a stepwise selection. A p-value <0.05 was considered statistically significant. All p-values are two-sided.

Due to the presence of missing data, the SMC-FCS imputation technique was used. The complete case analysis will be performed and considered as the main result, whilst the results obtained from the imputed dataset will be considered as sensitivity analysis. All the main analyses were performed using the statistical software SAS v. 9.4 (SAS Institute Inc., Cary, NC, USA), the imputation techniques and the sensitivity analysis were performed by using the software R (https://www.r-project.org/).

## Results

### Patients

**Figure 1:**
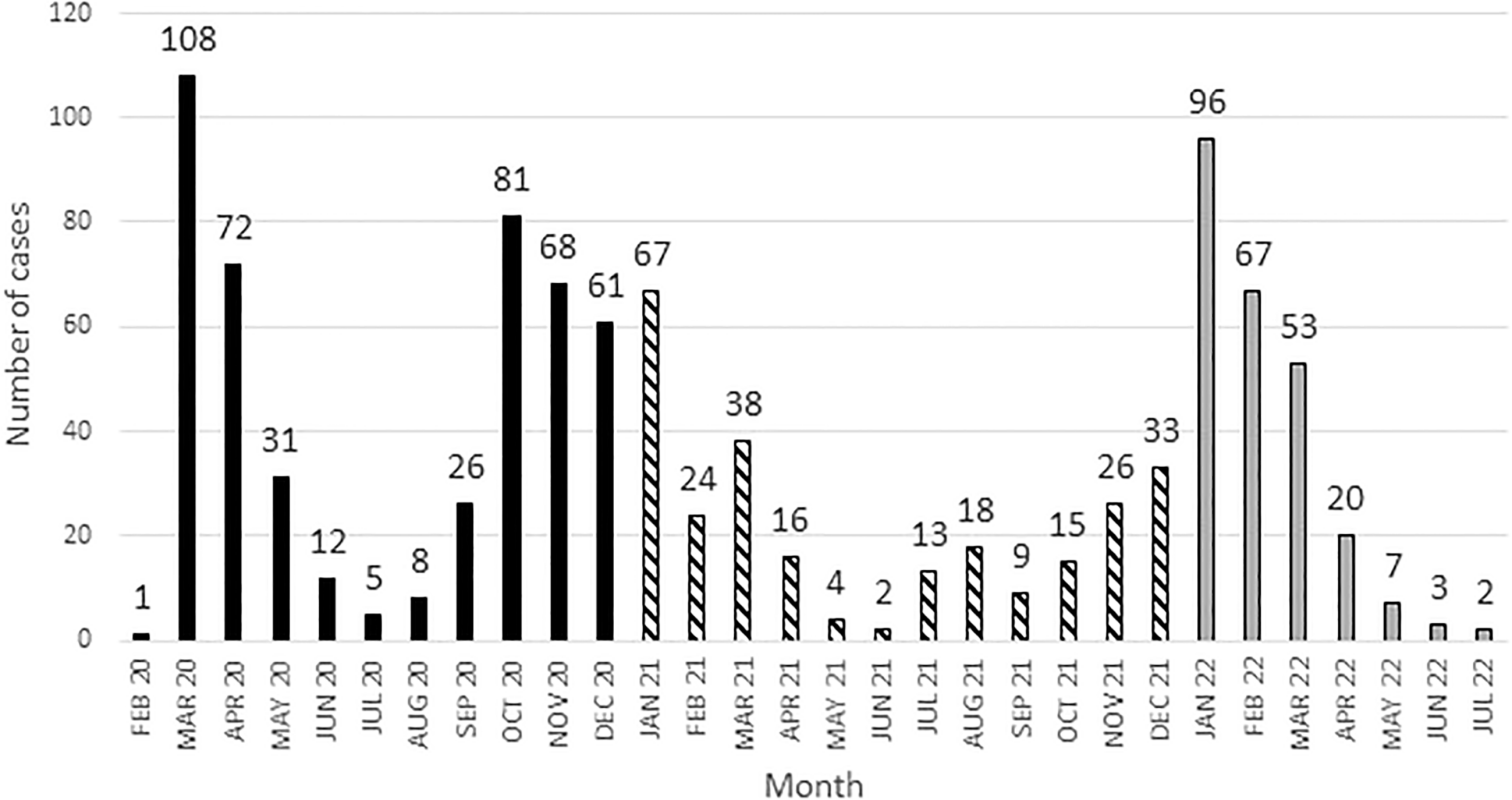
Distribution of cases over time.

986 patients from 22 countries fulfilled the criteria for inclusion. The median age was 50.3 years (min – max; 1.0 – 80.7) and did not differ over the years. One hundred fourteen patients were children (< 18 years of age; median age 10.5 (1.0 – 17.8)). The distribution of reported cases over time is shown in **Figure 1**. Details about donor type, conditioning, stem cell source, and GVHD are shown in **Table 1**.

**Table 1:** Characteristics of patients having undergone allogeneic HCT.

	Year			P-value
	2020	2021	2022	
	N= 473	N=265	N=248	
Median age at COVID-19 diagnosis	51.0 (1.0 - 80.4)	51.2 (1.3 - 80.7)	48.0 (2.2 - 75.8)	0.1
Sex	N (%)	N (%)	N (%)	
Male	271 (57.3)	157 (59.2)	146 (58.9)	0.9
Female	202 (42.7)	108 (40.8)	102 (41.1)	
CMV serostatus				0.001
R-/D-	75 (15.9)	50 (18.9)	56 (22.6)	
R-/D+	25 (5.3)	19 (7.2)	21 (8.5)	
R+/D-	95 (20.1)	70 (26.4)	74 (29.8)	
R+/D+ Mi	255 (53.9)	120 (45.3)	96 (38.7)	
Missing	23 (4.9)	6 (2.3)	1 (0.4)	
CMV serostatus in patients				
R-	104 (22.0)	70 (26.4)	77 (31.0)	0.03
R+	369 (78.0)	195 (73.6)	171 (69.0)	
Stem cell source				
BM (bone marrow)	76 (16.1)	49 (18.5)	88 (35.5)	<0.0001
PB (peripheral blood)	393 (83.1)	206 (77.7)	154 (62.1)	
CB (cord blood)	4 (0.8)	10 (3.8)	6 (2.4)	
HLA match				
Matched family	168 (35.5)	74 (27.9)	57 (23.0)	0.001
Mismatched family	73 (15.4)	28 (10.6)	35 (14.1)	
Unrelated	232 (49.0)	163 (61.5)	156 (62.9)	
Conditioning				
Myeloablative	246 (52.0)	135 (50.9)	162 (65.3)	0.001
Reduced	214 (45.2)	129 (48.7)	83 (33.5)	
Missing	13 (2.7)	1 (0.4)	3 (1.2)	
*In-vivo* T cell depletion				
No	235 (49.7)	120 (45.3)	131 (52.8)	0.2
Yes	235 (49.7)	145 (54.7)	115 (46.4)	
Missing	3 (0.6)	0 (0.0)	2 (0.8)	
aGvHD at time of COVID-19	33 (7.0)	18 (6.8)	13 (5.2)	0.8
Chronic GvHD at time of COVID-19				
No (never)	258 (58.6)	146 (56.8)	156 (65.8)	0.001
Yes, ongoing	133 (30.2)	82 (31.9)	44 (18.6)	
Resolved	26 (5.9)	13 (5.1)	3 (1.3)	
Missing	23 (5.2)	16 (6.2)	34 (14.3)	
Corticosteroids				
No	307 (64.9)	170 (64.2)	161 (64.9)	0.08
Yes	150 (31.7)	69 (26.0)	52 (21.0)	
Missing	16 (3.4)	26 (9.8)	35 (14.1)	
Immunosuppression				
No	189 (40.0)	120 (45.3)	114 (46.0)	0.01
Yes	269 (56.9)	121 (45.7)	100 (40.3)	
Missing	15 (3.2)	24 (9.1)	34 (13.7)	

### COVID-19

The median time from most recent HCT to diagnosis of COVID-19 was 20 months (min – max; 0.0 – 383.9). The median time was 19.3 (0.0 - 287.6) months during 2020, 21.2 (0.1 - 324.5) months during 2021, and 19.7 (0.1 – 383.9) months during 2022 (p = NS). At the time of diagnosis, fewer patients required oxygen support during the later years (17.3% during 2020, 11.0% during the 2021, and 0.9% during 2022). The proportions of patients with ongoing immunosuppression decreased over the periods (p=0.01). The proportion of the patients being hospitalized during the COVID-19 episode decreased over time with 56.0% hospitalized during 2020, 38.1% during 2021, and 15.9% during 2022 (p<.0001). The proportion of patients with lower respiratory tract disease decreased over time. This decrease was seen both at the time of COVID-19 diagnosis (12.5% in 2020; 10.9% in 2021; 2.8% in 2022) and during follow-up (50.5% in 2020; 30.9% in 2021; 15.7% in 2022). The proportion of patients treated with antiviral drugs or monoclonal antibodies increased over time as such became available (**Supplementary Table 2**).

### Previous vaccination

253 patients had been vaccinated before the diagnosis of COVID-19. 34 patients had received one dose, 77 two doses, 119 three doses, 22 four doses, and one patient had received five doses of vaccine. The absolute majority had received mRNA vaccines. Only two of 204 (1%) fully vaccinated patients (defined as having received at least two doses with the 2^nd^ dose given > 14 days before diagnosis of COVID-19) died from COVID-19 while five patients died from other causes.

### Overall survival in COVID-19

**Figure 2:**
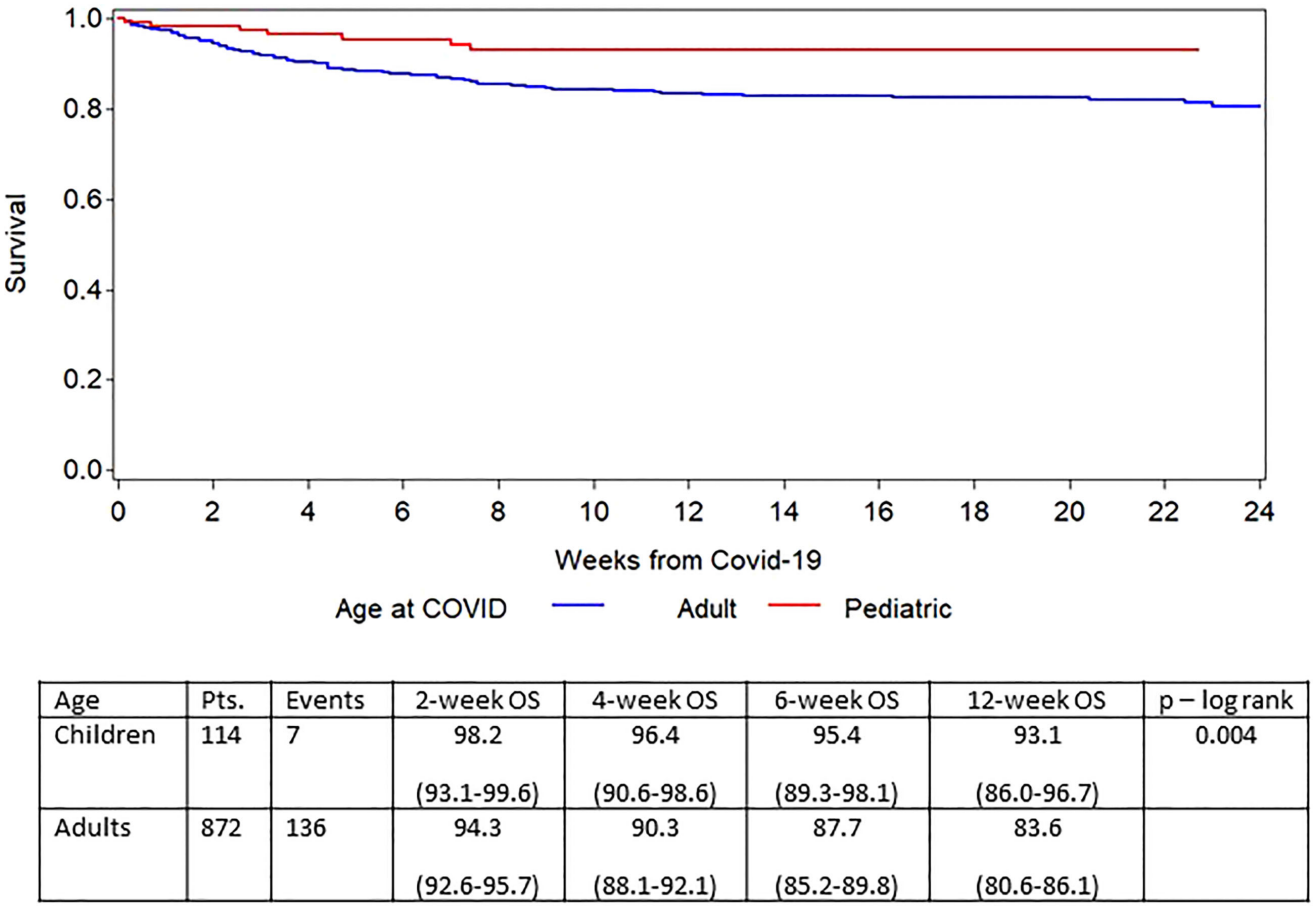
Survival after diagnosis of COVID-19 in adults and children.

**Figure 3:**
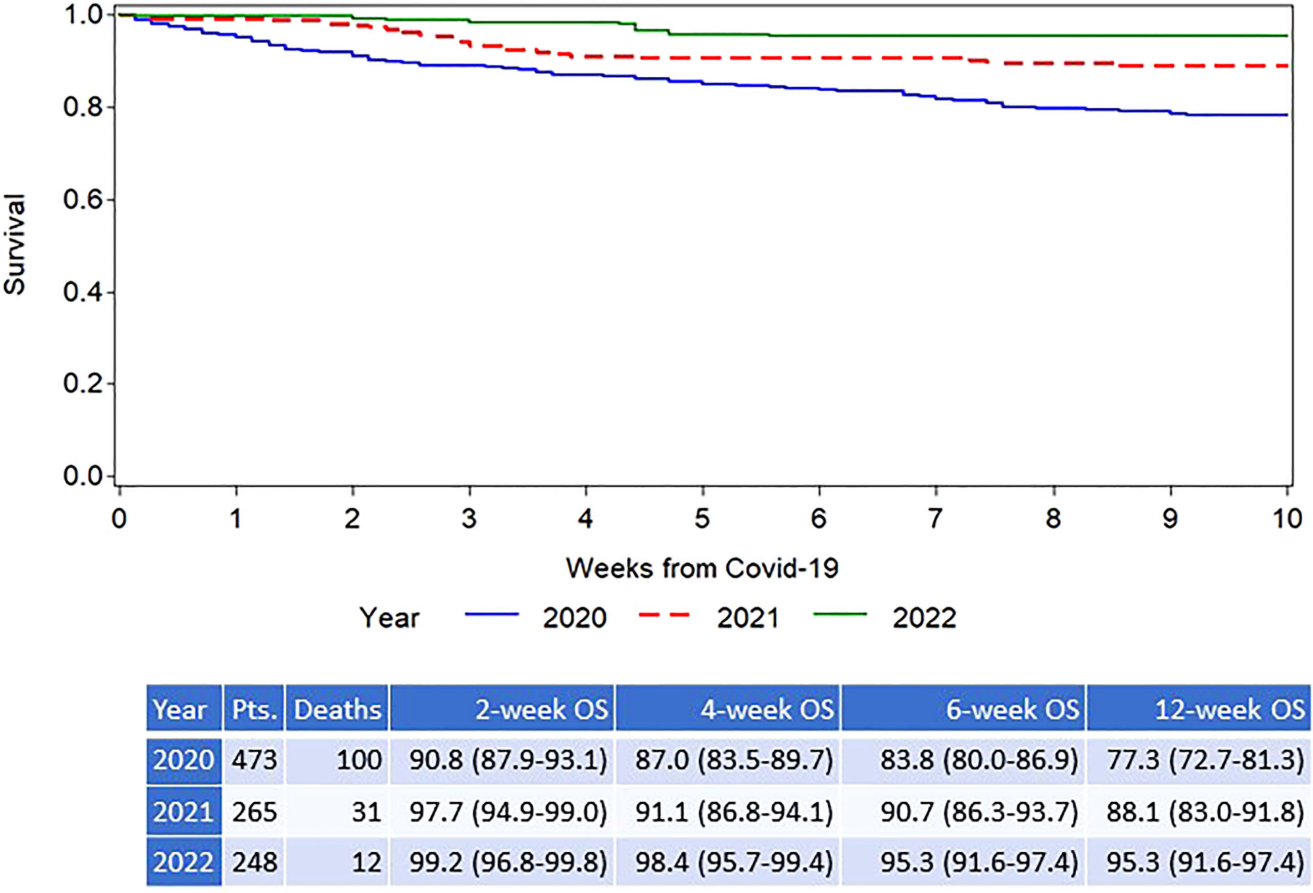
Overall survival after diagnosis of COVID-19 infection in allogeneic HCT recipients; split by calendar year.

**Figure 4:**
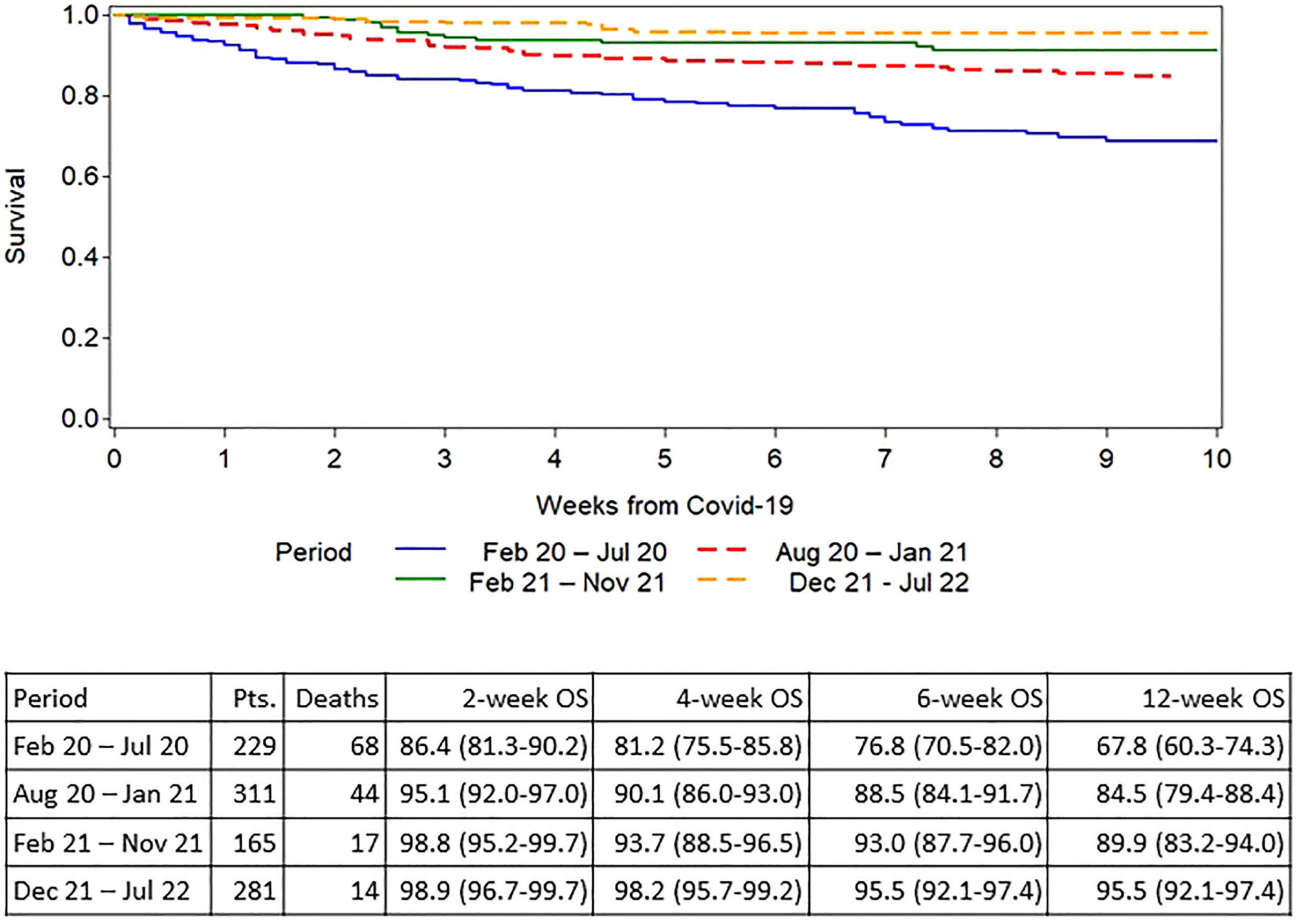
Overall survival after diagnosis of COVID-19 in allogeneic HCT recipients split by pandemic phase.

At the time of analysis, 145/986 (14.7%) patients had died; 124 patients (12.6%) died from COVID-19 and 21 patients died from other causes. The outcome was significantly better in children (**Figure 2**), who had a 6-week overall survival of 96.4% (95% CI 89.3% - 98.1%) compared to adults (6-week OS of 87.7% (95% CI 85.2% - 89.8%; P = .01). Survival curves for the three calendar years are shown in **Figure 3** showing an improvement in overall survival from 83.8% (95% CI 80.0-86.9) in 2020 to 90.7% (95% CI 86.3-93.7) during 2021, and 95.3% (95% CI 91.6% - 97.4%) during 2022 (p<.0001). Similar differences were seen when survival was analysed based on the different phases of the pandemic (**Figure 4**). During the first phase (February – July 2020), the survival was 76.8% (95% CI 70.5-82.0. During the second phase before vaccines became available (August 2020 – January 2021), the survival was 88.5% (95% CI 84.1-91.7). The survival had improved further during the phase when the vaccines were introduced also coinciding with the rapid spread of the Delta variant (93.0% (95% CI 87.7-96.0)) and finally the best overall survival was seen when the Omicron variant had become dominant (95.5% (95% CI 92.1-97.4)). The risk for developing lower respiratory tract disease also decreased over time both at the time of diagnosis and during follow-up supporting a lower severity of SARS-CoV-2 infections with time.

Univariate risk factors for survival are shown in **Supplementary Table 1**. Risk factors identified in multivariate analysis for overall survival are shown in **Table 2**. Increasing age, worse performance status at allo-HCT, contracting COVID-19 a short time after allo HCT especially during the first 30 days after HCT, ongoing immunosuppressive therapy, pre-existing lung pathology, and being CMV seropositive all had negative impact on overall survival while patients contracting COVID-19 in 2021 or 2022 had improved survival. Furthermore, ongoing corticosteroids at diagnosis of COVID-19 had also a negative impact HR 2.69 (95% CI 1.84 – 3.93) but overlapped strongly with ongoing immunosuppression and was therefore not included in the final multivariate model. In addition, we identified in univariate analysis low absolute neutrophil count <0.5 x10^9^/L (p<.0001), and absolute lymphocyte count < 0.2 x 10^9^/L (p<0.006) as significant risk factors. However, due to missing data, these variables could not be included in the multivariate analysis. Factors with no impact on OS in univariate analyses were patient sex, GVHD, underlying diagnosis, and country (data not shown).

**Table 2:** Overall survival – Multivariate analysis.

Variables		CCA (n=792)		Imputed - SMC-FCS (n=986)	
		HR (95% C.I.)	p	HR (95% C.I.)	p
Age at COVID	Continuous (10-yr effect)	1.31 (1.16-1.47)	<0.0001	1.33 (1.20-1.48)	<0.0001
Time after HSCT	> 100 days	1.00	<0.0001*	1.00	<0.0001*
	31-100	1.84 (1.05-3.23)	0.03	2.11 (1.29-3.43)	0.003
	<30 days	5.21 (2.81-9.63)	<0.0001	5.13 (2.91-9.02)	<0.0001
CMV serostatus patient	Negative	1.00		1.00	
	Positive	2.38 (1.35-4.20)	0.003	2.02 (1.26-3.25)	0.004
Performance status (Karnofsky/Lansky)	>=90	1.00		1.00	
	<90	3.09 (2.06-4.64)	<0.0001	3.16 (2.10-4.76)	<0.0001
Ongoing immune suppressive therapy	No	1.00		1.00	
	Yes	2.18 (1.34-3.56)	0.002	1.90 (1.23-2.93)	0.004
Year	2020	2.89 (1.48-5.61)	0.002	3.77 (2.03-6.99)	<0.0001
	2021	1.56 (0.72-3.37)	0.3	2.18 (1.10-4.32)	0.027
	2022	1.00	<0.0001*	1.00	<0.0001*
Other lung pathology	No	1.00		1.00	
	Yes	2.47 (1.47-4.14)	0.0006	2.15 (1.32-3.50)	0.003

CCA, Complete Case Analysis.

SMC-FCS, Substantive-model Compatible Fully Conditional Specification.

*Overall comparison.

**Figure 5:**
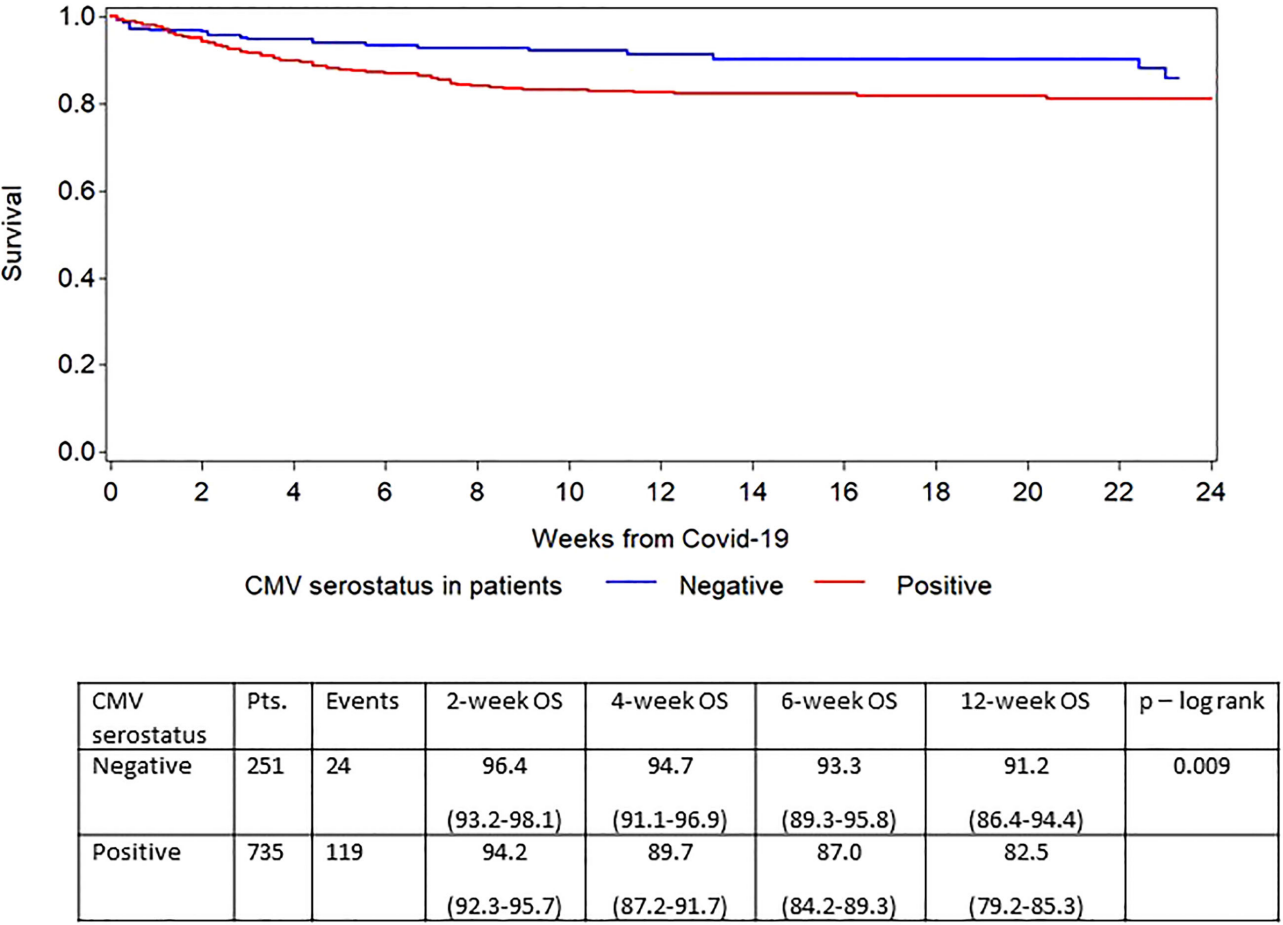
Survival based on recipient CMV serostatus.

The finding that CMV seropositivity in the recipient had a negative impact prompted us to study this risk factor in detail. **Figure 5** shows the Kaplan-Meier survival curves for CMV seropositive and CMV seronegative patients, respectively. The overall survival improved over time for both CMV seropositive and CMV seronegative patients but more strongly among the CMV seropositive patients (**Table 3**). We also looked at the effect on survival for the different donor/recipient CMV serostatus combinations. Compared to HCT with donor and recipient both seronegative as baseline, there was no impact of a negative recipient having a CMV seropositive donor and furthermore there was no additional negative effect of having a CMV seronegative donor to a CMV seropositive patient compared to both being CMV seropositive. Thus, it is the serostatus of the patient independent of the donor that mediates the negative effect.

**Table 3:** Effect of CMV serostatus on overall survival for the different periods.

	Patients	Deaths	2-week OS	4-week OS	6-week OS	12-week OS
**CMV serostatus (R/D)**						
R-/D-	181	20	95.0 (90.6-97.4)	93.3 (88.5-96.1)	91.4 (86.0-94.7)	90.6 (85.0-94.1)
R-/D+	65	3	100.0	100.0	100.0	93.9 (77.7-98.4)
R+/D-	239	36	95.8 (92.3-97.7)	91.4 (86.9-94.3)	88.0 (83.0-91.7)	85.3 (79.7-89.5)
R+/D+	471	81	93.3 (90.7-95.3)	88.5 (85.2-91.1)	86.0 (82.3-88.9)	80.7 (76.3-84.3)
						P= 0.03
**Patient CMV serostatus**						
Negative	251	24	96.4 (93.2-98.1)	94.7 (91.1-96.9)	93.3 (89.3-95.8)	91.2 (86.4-94.4)
Positive	735	119	94.2 (92.3-95.7)	89.7 (87.2-91.7)	87.0 (84.2-89.3)	82.5 (79.2-85.3)
						P=0.009
**Year 2020**						
R CMV Negative	104	15	93.3 (86.4-96.7)	92.3 (85.2-96.1)	91.0 (83.4-95.2)	86.0 (76.1-92.0)
R CMV Positive	369	85	90.2 (86.6-92.8)	85.4 (81.3-88.7)	81.7 (77.2-85.4)	74.8 (69.4-79.4)
**Year 2021**						
R CMV Negative	70	7	*98.6* *(90.3-99.8)*	*93.8* *(84.4-97.6)*	*92.1* *(81.9-96.6)*	*92.1* *(81.9-96.6)*
R CMV Positive	195	24	*97.4* *(93.8-98.9)*	*90.2* *(84.9-93.7)*	*90.2* *(84.9-93.7)*	*86.7* *(80.2-91.1)*
**Year 2022**						
R CMV Negative	77	2	*98.7* *(91.1-99.8)*	*98.7* *(91.1-99.8)*	*97.3* *(89.7-99.3)*	*97.3* *(89.7-99.3)*
R CMV Positive	171	10	*99.4* *(95.9-99.9)*	*98.2* *(94.5-99.4)*	*94.3* *(89.4-97.0)*	*94.3* *(89.4-97.0)*

## Discussion

The emergence of SARS-CoV-2 and COVID-19 has had a very strong impact on transplant patients with several reports showing high risks for morbidity and mortality especially during the first months after the emergence of the virus. In a previous report based on the EBMT registry COVID-19 resulted in an attributable mortality of 25% (2). We found a 6-week probability of survival of 77.9% and 72.1% in allogeneic and autologous HCT recipients, respectively (2). The CIBMTR published results on 314 patients (3). Their 30-day survival after diagnosis of COVID-19 was 68% among allogeneic HCT recipients and 67% among autologous HCT recipients. They found age over 50, male sex, and time from HCT to COVID-19 diagnosis of less than a year to be significant risk factors for mortality among allogeneic recipients. Other studies of HCT patients have reported similar outcomes (4–12). Since the early reports, there have been major developments especially with the introduction of vaccines and therapies of COVID-19 but also the spread of Delta and the Omicron variant including many sub-variants. This has resulted in lower mortality in patients with hematological malignancies including allogeneic HCT recipients (13–15), but no large analysis has been performed regarding risk factors and changes in outcome over time in this patient group.

In this report, including by far the largest cohort of allo-HCT recipients analysed until now, we report a successively improved outcome from the initially reported 76.8% 6-week survival to a survival of 95.% during the Omicron phase of the pandemic. Furthermore, the proportion of patients hospitalized and the proportion of patients developing lower respiratory tract disease decreased with time supporting a lower severity of SARS-CoV-2 infection over time.

The patients included in the first phase and here used as a comparator are the same as reported in our previous study (2). The way of collecting data remained the same although the CRFs have been updated to include some new variables over time especially regarding vaccination status, but it can’t be ascertained that the willingness of the centers to report was the same over time as we learned more and more about COVID-19 and its impact on allogeneic HCT recipients.

In our previous study, risk factors having significant impact on overall survival were increasing patient age and poorer performance status. These risk factors remained significant over time in the different pandemic phases and especially children and adolescents continued to do significantly better. Age has been an important factor in most reports on COVID-19 in the general population (16–18). Poor performance status is usually due to comorbidities such as extensive GVHD, but such a correlation was not found this time even in the extended cohort. New risk factors identified for fatal COVID-19 when controlled for year of COVID-19 were being infected the first 100 days and especially the first 30 days after HCT, ongoing immunosuppression, and pre-existing lung pathology. All these were expected considering the impact of other viral infections on early post-transplant morbidity and mortality.

We were surprised in our previous analysis that the time from HCT did not impact overall survival and neither did ongoing immunosuppression nor the presence of GVHD. One question was if these factors would have an impact when analysing a larger cohort and such was indeed the case in this extended cohort. Patients contracting COVID-19 the first 30 days after the HCT had a more than fivefold increase in the HR for death while there was an almost two-fold risk increase during the 30 – 100 days period compared to patients diagnosed with COVID-19 later (p<.0001). It is, however, possible that the later the patient was after transplant, the less likely it was for a patient to be reported to the study especially if the infections were mild. Thus, this effect of time might be even stronger for all SARS-CoV-2 infected allo-HCT patients than what we found. In addition, ongoing immune suppression was also an independent negative factor for survival and immunosuppression is given mostly to patients quite early after HCT. Another possible influencing factor is that patients respond poorly to vaccination performed early after HCT (19–22). All this taken together supports the need for protecting the most vulnerable patients from becoming infected. This high mortality in patients early after transplantation clearly supports the necessity for continued vigilance in the infection control efforts at hospitals performing allo-HCT in particular keeping transplant units as far as possible free from COVID-19 for example by systematic PCR screening and early antiviral therapy.

We also found that CMV serostatus impacted on the risk for dying within 6-weeks after contracting SARS-CoV-2 infection. CMV seropositivity has been reported to increase the risk for hospitalization in patients infected with SARS-CoV-2 (23). Furthermore, CMV seropositivity has been proposed as one possible reason for the severe impact of COVID-19 in the elderly and lower impact in children (24, 25). CMV is a major pathogen after allogeneic HCT and seropositive patients have a decreased overall survival rate and an increased risk of non-relapse mortality after allogeneic HCT (26, 27). CMV can also influence many different immune system functions including those of NK-cells and T cells. We found in this analysis that CMV serostatus has an independent negative effect on overall survival when corrected for other risk factors including year of SARS-CoV-2 infection adding to existing data about the negative effect of CMV on the seriousness of COVID-19. The negative impact existed over time although the survival improved over time in both seropositive and CMV seronegative recipients. Recently Perera et al. showed that patients with severe COVID-19 had higher levels of CMV-specific IgG at the time of COVID-19 (28). Furthermore, they showed in an *in vitro* study that CMV increases the number of cells infected by SARS-CoV-2 and upregulates the SARS-CoV-2 receptor ACE2, the SARS-CoV-2 cell entry receptor. It is possible that the negative effect is through local CMV replication in the airways We don’t have information on either letermovir prophylaxis or CMV reactivations so we can only speculate about the effects of possible interventions.

We could not find an independent effect of donor serostatus although the number of CMV seronegative patients having received grafts from CMV seropositive individuals was low and even fewer such patients were infected with SARS-CoV-2 during the most vulnerable early period.

The design of this study does not allow to look at the possible protective effects of vaccination since patients fully protected against infection would not have been reported to our registry. However, the rate of fatal COVID-19 among fully vaccinated patients was only 1% strongly supporting high effectiveness against severe COVID-19. This is in agreement with a previous report showing that additional doses of vaccine can improve outcome in patients with hematological malignancies (13). As could be expected, the proportion of patients treated with either monoclonal antibodies or antiviral agents increased with time and this can have improved the outcome of COVID-19.

We conclude that the mortality in and severity of COVID-19 have decreased over time during the pandemic. We have identified several risk factors for mortality including the previously known age and comorbidity. Newly identified risk factors are short time after allogeneic HCT, ongoing immunosuppression, and interestingly CMV seropositive patient status. Additional studies have to be performed to investigate the role of CMV reactivation in outcome of COVID-19

## Data availability statement

The original contributions presented in the study are included in the article/**Supplementary Material**. A request can be made to the chairperson of the Infectious Diseases Working Party of the EBMT. Requests to access the datasets should be directed to idwpebmt@lumc.nl.

## Ethics statement

The studies involving human participants were reviewed and approved by Swedish Ethics Review Authority. Written informed consent for their data to be reported to the EBMT registry was provided by each participant or their legal guardian.

## Author contributions

PL, RC, JLP, JS, MM designed the study and worked as a writing committee. GT is the study statistician. NKn managed the registry data. NKr, DA, SC critically reviewed the manuscript. All other authors submitted cases to the study and critically reviewed the manuscript. All authors contributed to the article and approved the submitted version.
