# Supplementary material for: Improved outcomes over time and higher mortality in CMV seropositive allogeneic stem cell transplantation patients with COVID-19; An infectious disease working party study from the European Society for Blood and Marrow Transplantation registry
Source: Front Immunol. 2023 Mar 7;14:1125824. doi: 10.3389/fimmu.2023.1125824 (PMC10028143; doi:10.3389/fimmu.2023.1125824)
Supplement: Supplementary file 1 [file Table_1.docx]

Supplementary informatinon

Supplementary table 1. Univariate analysis

|  |  | Patients | Deaths | Univariate analysis | |
| --- | --- | --- | --- | --- | --- |
| Variable |  |  |  | HR (95% C.I.) | p |
| Sex | Male | 574 | 89 | 1.00 |  |
|  | Female | 412 | 54 | 0.86 (0.61-1.20) | 0.4 |
|  |  |  |  |  |  |
| Age at covid | Continuous  (10-yr effect) | 986 | 143 | 1.40 (1.26-1.56) | <0.0001 |
|  |  |  |  |  |  |
| Age at covid | Adult | 872 | 136 | 2.89 (1.35-6.18) | 0.006 |
|  | Children | 114 | 7 | 1.00 |  |
|  |  |  |  |  |  |
| Diagnosis | AML/ALL | 501 | 73 | 1.00 | 0.4 |
|  | CML/MDS/MPN | 254 | 41 | 1.10 (0.75-1.62) |  |
|  | NHL/Hodgkin/CLL | 112 | 18 | 1.18 (0.70-1.98) |  |
|  | Other | 119 | 11 | 0.63 (0.34-1.19) |  |
|  |  |  |  |  |  |
| Time from most recent transplant to covid | < 1 year | 358 | 84 | 2.70 (1.94-3.77) | <0.0001 |
|  | >= 1 year | 628 | 59 |  |  |
|  |  |  |  |  |  |
| Time from most recent transplant to covid | <30 days | 36 | 16 | 4.31 (2.54-7.29) | <0.0001 |
|  | 31-100 | 89 | 22 | 2.10 (1.32-3.32) |  |
|  | >100 days | 861 | 105 | 1.00 |  |
|  |  |  |  |  |  |
| CMV serostatus patient | Negative | 251 | 24 | 1.00 |  |
|  | Positive | 735 | 119 | 1.78 (1.15-2.76) | 0.01 |
|  |  |  |  |  |  |
| Performance status (Karnofsky/Lansky) | 10-point effect | 847 | 123 | 0.60 (0.55-0.65) | <0.0001 |
|  | ≥90 | 582 | 44 | 1.00 |  |
|  | <90 | 265 | 79 | 4.16 (2.88-6.02) | <0.0001 |
|  |  |  |  |  |  |
| Ongoing immunosuppression (no steroids) | No | 694 | 92 | 1.00 |  |
|  | Yes | 219 | 36 | 1.37 (0.93-2.01) | 0.11 |
|  |  |  |  |  |  |
| Ongoing steroids | No | 638 | 62 | 1.00 |  |
|  | Yes | 271 | 64 | 2.41 (1.70-3.42) | <0.0001 |
|  |  |  |  |  |  |
| Ongoing IS therapy | No | 423 | 28 | 1.00 |  |
|  | Yes | 490 | 100 | 3.28 (2.16-4.98) | <0.0001 |
|  |  |  |  |  |  |
| GvHD | No | 611 | 74 | 1.00 |  |
|  | Yes | 303 | 51 | 1.38 (0.97-1.97) | 0.08 |
|  |  |  |  |  |  |
| BOS | No | 859 | 114 | 1.00 | 0.12 |
|  | Yes | 47 | 10 | 1.68 (0.88-3.21) |  |
|  |  |  |  |  |  |
| Other lung pathology | No | 812 | 98 | 1.00 |  |
|  | Yes | 70 | 22 | 2.92 (1.84-4.63) | <0.0001 |
|  |  |  |  |  |  |
| BOS or other lung pathology | No | 777 | 94 | 1.00 |  |
|  | Yes | 107 | 28 | 2.34 (1.53-3.56) | <0.0001 |
|  |  |  |  |  |  |
| ANC | <500 | 42 | 21 | 3.31 (2.06-5.32) | <0.0001 |
|  | >=500 | 602 | 94 | 1.00 |  |
|  |  |  |  |  |  |
| ALC | <200 | 49 | 20 | 2.54 (1.57-4.13) | 0.0002 |
|  | >=200 | 581 | 91 |  |  |
|  |  |  |  |  |  |
| Neutrophil/lymphocyte ratio | <median (2.56) | 309 | 44 | 1.00 |  |
|  | >= median (2.56) | 310 | 62 | 1.31 (0.89-1.93) | 0.17 |
|  |  |  |  |  |  |
| Lymphocyte/CRP ratio | <median (0.07) | 257 | 72 | 1.00 |  |
|  | >= median (0.07) | 258 | 24 | 0.33 (0.21-0.52) | <0.0001 |
|  |  |  |  |  |  |
| Wave | February 2020 – July 2020 | 229 | 68 | 6.67 (3.75-11.88) | <0.0001 |
|  | August 2020 – January 2021 | 311 | 44 | 2.82 (1.54-5.15) |  |
|  | February 2021 – November 2021 | 165 | 17 | 1.93 (0.95-3.92) |  |
|  | December 2021 - July 2022 | 281 | 14 | 1.00 |  |
|  |  |  |  |  |  |
| Year | 2020 | 473 | 100 | 4.55 (2.50-8.29) | <0.0001 |
|  | 2021 | 265 | 31 | 2.35 (1.21-4.59) |  |
|  | 2022 | 248 | 12 | 1.00 |  |
|  |  |  |  |  |  |
| Country | Spain | 108 | 17 | 1.00 | 0.2 |
|  | Italy-UK | 295 | 48 | 1.00 (0.58-1.75) |  |
|  | Other | 583 | 78 | 0.75 (0.44-1.26) |  |
