# Supplementary material for: Improved outcomes over time and higher mortality in CMV seropositive allogeneic stem cell transplantation patients with COVID-19; An infectious disease working party study from the European Society for Blood and Marrow Transplantation registry
Source: Front Immunol. 2023 Mar 7;14:1125824. doi: 10.3389/fimmu.2023.1125824 (PMC10028143; doi:10.3389/fimmu.2023.1125824)
Supplement: Supplementary file 2 [file Table_2.docx]

Supplementary table 2. Use of antiviral drugs and monoclonals during the different years.

| Agent | 2020 | 2021 | 2022 |
| --- | --- | --- | --- |
| Remdesivir | 25/473 (5.9%) | 30/265 (11.3%) | 29/248 (11.7%) |
| Molnupiravir | 0 | 0 | 7/248 (2.8%) |
| Nirmatrelvir/ritonavir | 0 | 0 | 9/248 (3.6%) |
| Monoclonal antibodies | 0 | 46/219 (17.4%) | 75/248 (30.2%) |
